# Novel Soluble apxIVA-Truncated Protein and Its Application to Rapid Detection and Distinction of *Actinobacillus pleuropneumoniae* Wild-Strain-Infected Samples from Those Vaccinated with *apxIV*-Partially Deleted Vaccine

**DOI:** 10.3390/vetsci12030278

**Published:** 2025-03-16

**Authors:** Jing Rao, Xiaoyu Liu, Xi Zhu, Yongle Qi, Huanchun Chen, Weicheng Bei

**Affiliations:** 1 National Key Laboratory of Agricultural Microbial Resources Discovery and Utilization, Huazhong Agricultural University, Wuhan 430070, China; raojingok@webmail.hzau.edu.cn (J.R.); liuxiaomei@webmail.hzau.edu.cn (X.L.); zxzxzzx@webmail.hzau.edu.cn (X.Z.); qyl@webmail.hzau.edu.cn (Y.Q.); chenhch@mail.hzau.edu.cn (H.C.); 2 The Cooperative Innovation Center for Sustainable Pig Production, Huazhong Agricultural University, Wuhan 430070, China; 3 Hubei Hongshan Laboratory, Huazhong Agricultural University, Wuhan 430070, China

**Keywords:** *Actinobacillus pleuropneumoniae*, apxIV, wild strain, gene-deleted vaccine, ELISA, colloidal gold immunochromatographic strip, rapid detection

## Abstract

*Actinobacillus pleuropneumoniae* (APP) is a bacterial pathogen causing porcine pleuropneumonia, causing great economic loss to the global pig industry. Although natural apxIV contributes to the prevention and control of porcine pleuropneumonia, its isolation poses a great challenge, and recombinant soluble apxIV proteins tend to carry large molecular weight tags. The traditional serologic methods tend not to accurately detect the *apxIV*-partially deleted vaccine (GDV). In this study, we screened the soluble protein apxIVA N2 (756 bp) from six apxIV-truncated proteins and applied it to the enzyme-linked immunosorbent assay (ELISA) and colloidal gold immunochromatographic strip for detecting the samples vaccinated with APP GDV. The results indicate that N2 was close to the natural apxIV protein in terms of structure and function as it only contained a single His (0.86 kDa) tag and a single S (2 kDa) tag. Among the six candidate proteins, N2 exhibited the best performance in distinguishing APP-infected samples from those vaccinated with the APP GDV. Both ELISA and colloidal gold immunochromatographic strips based on this protein exhibited an excellent performance in detecting and distinguishing wild-strain-infected samples from those vaccinated with the subunit vaccine or the GDV. In addition, three monoclonal antibodies against different antigenic epitopes were identified using these truncated proteins. Our studies are of great significance for further research on APP, the differential diagnosis of wild strains and vaccine strains, and pig control breeding, exhibiting a broad application prospect in the on-site diagnosis of APP, particularly in remote areas lacking detection instruments and professionals.

## Simple Summary

Porcine pleuropneumonia, which is caused by *Actinobacillus pleuropneumoniae*, represents a significant threat to the global pig farming industry. Accurately diagnosing this disease is challenging, particularly when distinguishing between natural infections and those resulting from the *apxIV*-partially deleted vaccine. In this study, we identified a soluble recombinant apxIVA N2 protein, which carries only small molecular tags (a single His tag and a single S tag), and developed two diagnostic methods based on this protein to address the issue. Due to the small molecular tags, this protein closely resembles the natural protein in both structure and function, while also offering advantages in terms of expression yield, solubility, and cost-effectiveness. The two methods we developed—enzyme-linked immunosorbent assay and colloidal gold immunochromatographic strip—successfully differentiated between pigs infected with the wild strain and those vaccinated with the gene-deleted vaccine, providing a reliable tool for distinguishing between the two groups. Additionally, we screened three monoclonal antibodies specific to different antigenic epitopes. Our study has significant implications for further studies on porcine pleuropneumonia, differential diagnosis of wild and vaccine strains, and pig breeding control. It also holds broad application potential, particularly in remote areas with limited access to diagnostic tools and professionals.

## 1. Introduction

Porcine pleuropneumonia (PCP) is a severe bacterial respiratory disease caused by *Actinobacillus pleuropneumoniae* (APP), which is highly prevalent in pig farms worldwide [1]. PCP is mainly characterized by acute hemorrhagic fibrinous pneumonia and chronic fibrinous necrotizing pleuropneumonia. Acutely infected pigs often bleed from the mouth and nose and die suddenly, while chronically infected pigs often become a hidden source of infection, and they can continuously spread the pathogen. Pigs carrying the APP pathogen stay in a sub-healthy state for a long time, in which they are susceptible to other infections [2,3,4]. APP infection often leads to slow growth and even the death of pigs, thus causing huge economic losses to the global pig industry [5].

Antibiotics used to be considered as the optimal measure to control APP. However, untargeted, excessive use of antibiotics has resulted in the development of drug resistance over the decades, which has prompted a search for new strategies [6,7]. A variety of novel vaccines have been employed for the prevention and control of APP in pig farms [8,9]. APP is classified into 19 serotypes according to differences in the capsular antigen, and the recombinant protein vaccines exhibit limited cross-protection effects against different serotypes of this pathogen [10,11]. APP-HB-04M (a gene-deleted vaccine, GDV), with resistance to several highly virulent serotype strains of APP, was developed in our previous study and has been widely used in some leading large-scale pig farms in China, exhibiting satisfactory results. APP is mainly transmitted among pigs by direct or indirect contact (aerosols and pollutants), and the infection symptoms of APP are similar to those of other porcine respiratory pathogens such as *Streptococcus suis* and *Pasteurella multocida*, making it difficult to distinguish different pathogens by visual inspection and with diagnostic experience [12,13]. Since all 19 serotypes of APP contain the apxIV protein, the current diagnostic methods primarily target apxIV, including both serological and molecular biological methods. However, molecular methods, represented by PCR and qPCR, have several drawbacks, including overreliance on nucleic acid extraction instruments, complex operational procedures, high requirements for experimental techniques and environmental conditions, and potential errors due to temperature-sensitive amplification. Furthermore, these methods require large, costly equipment, which makes them impractical for rapid field diagnosis and transportation, especially in resource-limited settings [14]. Serological diagnostic methods require no large equipment as they rely on noncovalent interaction between the antigen and antibody, thus simplifying detection procedures, improving detection efficiency, and making the transportation of serological diagnostic instruments more convenient, especially in remote areas where resident veterinarians and veterinary workstations are scarce [15]. ELISA is the most classical method used in the serological diagnosis of APP, and the absence of apxIV in recombinant protein vaccines enables the existing ELISA methods to distinguish the samples infected with wild strain from those vaccinated with recombinant protein vaccines [16]. However, because of the difference in the selection of target fragments, existing ELISA kits may fail to distinguish the samples infected with wild strain from those vaccinated with APP GDV (APP-HB-04M) [17]. Additionally, no commercially available colloidal gold immunochromatographic strip exists for this purpose, likely due to the lack of suitable proteins.

Natural apxIV is secreted in low amounts only after the strain infects pigs, making the isolation of this protein difficult. Most recombinant apxIV proteins are expressed in inclusion bodies, and the remaining soluble apxIV proteins tend to carry large tags whose molecular weight may be even greater than that of the target protein, which makes their structures and functions different from those of natural apxIV proteins [18]. Furthermore, Large-molecular-weight tags pose an additional burden on biological protein synthesis, reducing the expression and quality of the target protein [19]. Small-molecular-weight tags have a weaker interference effect on the structure and function of the target protein, and thus these target proteins can maintain their natural properties. Studies based on proteins carrying small-molecular-weight tags can provide more reliable data [20]. Therefore, recombinant apxIV proteins that are close to the natural ones in structure and function are badly needed. Constructing apxIV proteins with small-molecular-weight tags or no tags is of great importance for revealing the pathogenic mechanisms of APP, developing drugs, and optimizing diagnostic methods.

**Figure 1:**
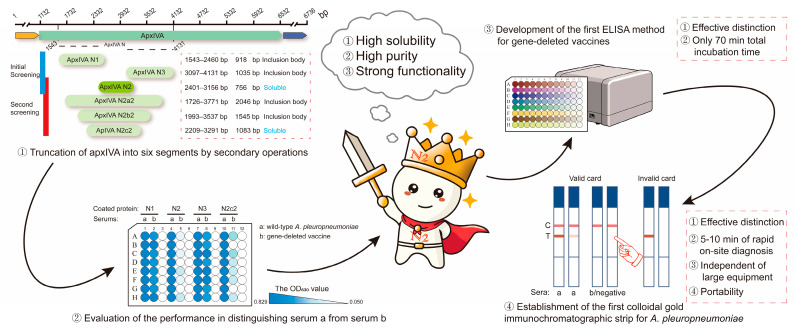
Schematic of the novel truncated protein and its application in distinguishing wild-type strain infection from vaccine immunization.

**Figure 2:**
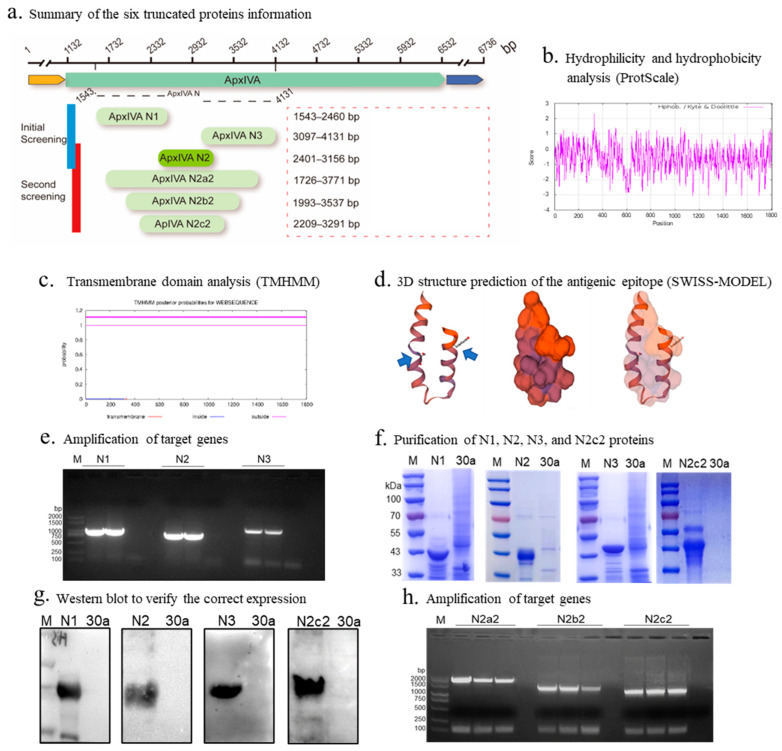
Truncation and expression of apxIVA. (**a**) Summary of the six truncated proteins’ information. (**b**) Hydrophilicity and hydrophobicity analysis of apxIV protein (ProtScale). (**c**) Transmembrane domain analysis of apxIV protein (TMHMM). (**d**) Three-dimensional structural prediction of the antigenic epitope of apxIV protein (SWISS-MODEL). (**e**) Amplification of target genes. (**f**) Purification of N1, N2, N3, and N2c2 proteins. (**g**) Western blot to verify the correct expression of N1, N2, N3, and N2c2 proteins. (**h**) Amplification of target genes.

Therefore, in this study, a truncated protein, apxIVA N2 (756 bp), was obtained after six-segment truncation. This protein was closer to the natural apxIV protein in structure and function since it contained only a single His (0.86 kDa) tag and a single S (2 kDa) tag. Subsequently, an ELISA kit and a colloidal gold immunochromatographic strip were developed based on this apxIVA N2 recombinant protein (Figure 1 and Figure 2a). This study lays a foundation for further research on APP and provides a valuable reference for the differential diagnosis of vaccine strains.

## 2. Materials and Methods

Sources of serum samples. APP-positive pig sera were purchased from Keqian Biological Co. Ltd. (Wuhan, China), and SPF-antibody-negative sera were purchased from Hongquan Biotechnology Co. Ltd. (Guangzhou, China). Keqian Biological Co. had previously tested the positive sera, and the results showed strong positivity. This positive serum was used as the positive control in all experiments of this study. Clinical serum samples were collected from the pig farms. The sera from pigs vaccinated with APP-HB-04M (gene-deleted vaccine, GDV) and collected at 21 days after the second immunization of 70-day-old pigs were stored in our laboratory.

Strain and primer design. The APP serovar 1 strain 4074 (GenBank:AF021919) used in this study was from the laboratory, and the primers were designed according to the nucleotide sequence of the *ApxIV* gene of this strain (Snapgene, v5.2). This strain was selected because most apxIV-related studies are based on this strain. Six fragments were selected through bioinformatics analysis using ProtScale, TMHMM, and SWISS-MODEL. The amplification fragment sites and amplification conditions are shown in Figure 2a and Table S1, and the remaining information on the PCR test is shown in the product manual.

Plasmid construction and protein expression. The pET-30a (+) vector contains both a polyhistidine tag (His tag) and an S tag to enhance protein expression and purification. The His tag facilitates efficient purification through immobilized metal affinity chromatography (IMAC), while the S tag—a short peptide sequence (KETAAAKFERQHMDS) derived from the N-terminus of pancreatic ribonuclease A (RNase A)—improves protein solubility and provides increased flexibility for detection via antibody binding. The 6 truncated genes were respectively cloned into the pET-30a (+) vector to obtain recombinant plasmids, and these recombinant plasmids were then transformed into competent *E. coli* BL21 (DE3) cells, and these cells were induced by 1 mM isopropyl-β-thio-galactopyranoside (IPTG) at 16 °C for 16 h. The empty vector, pET-30a (+), was used as the control. The bacterial pellets were harvested and resuspended in phosphate-buffered saline (PBS). After ultrasonication, broken bacterial supernatants and precipitates were collected for SDS-PAGE and Western blot. N2 and N2c2 proteins from the broken bacterial supernatants were further purified by Ni-NTA agarose beads. The N1 and N3 inclusion bodies were further purified by sodium N-lauroyl-sarcosinate, also known as sarkosyl (SKL). Finally, the purified proteins were divided into small aliquots and stored at −80 °C for subsequent experiments.

Establishment of ELISA of recombinant proteins. Briefly, we determined the optimal encapsulation concentration of each candidate protein and dilution folds of the corresponding serum via the checkerboard titration method using both SPF-antibody-positive and SPF-antibody-negative sera. To ensure the objectivity of the results, three biological replicates were performed for the establishment of the ELISA. Subsequently, the optimal conditions, such as the optimal blocking solution type, dilution solution type, primary antibody incubation time, secondary antibody incubation time, and 3,3′,5,5′-tetramethylbenzidine (TMB) incubation time, were determined using the variable control method. The optimal conditions for these methods were determined according to the highest P/N value (positive/negative), and the specific data under each condition are shown in Tables S2–S10. N2a2 and N2b2 were not selected as candidate proteins because they were inclusion bodies derived from the N2 protein extension.

Evaluation of immunogenicity and distinguishing performance of candidate proteins. ELISA methods for the N1, N2, N3, and N2c2 proteins were established, and APP-positive pig sera were employed to evaluate their immunogenicity. Furthermore, 10 sera from pigs vaccinated with the APP GDV were used to assess the performances of the candidate proteins to distinguish wild-strain-infected (positive) sera from the sera of pigs vaccinated with the APP GDV.

Evaluation of N2 ELISA. To determine the cut-off value for the N2 ELISA, 15 serum samples from pigs vaccinated with the APP GDV were analyzed. The cut-off value was calculated as the mean OD_630_ value of these 15 samples plus two times the standard deviation. Due to the unclear background of the serum samples from pig farms, the diagnostic sensitivity, diagnostic specificity, positive predictive value (PPV), and negative predictive value (NPV) of our method were calculated using the ApxIV-ELISA Antibody Detection Kit (Wuhan Keqian Biological Co., Ltd., Wuhan, China) results as the reference standard. The formulas are as follows: 1. Diagnostic Sensitivity = True Positives/(True Positives + False Negatives); 2. Diagnostic Specificity = True Negatives/(True Negatives + False Positives); 3. Positive Predictive Value (PPV) = True Positives/(True Positives + False Positives); 4. Negative Predictive Value (NPV) = True Negatives/(True Negatives + False Negatives). Additionally, the 95% confidence intervals (CIs) for each parameter were calculated using the Wilson Score Interval method to evaluate the statistical reliability of these performance metrics. Details of the biological replicates used in other performance evaluations are provided in the Results section and the corresponding supplementary tables.

Preparation of colloidal gold. A total of 10 mL of 1% chloroauric acid solution was added to 1 L of ultrapure water and heated until boiling. Two minutes after boiling, 10 mL of 1% trisodium citrate solution was added, and at this moment, the color of the solution gradually turned from black to wine red. The solution was boiled for 15 min, and then heating was terminated, and the solution was cooled down to room temperature to obtain a colloidal gold solution.

Preparation of colloidal gold conjugate and test strip. A total of 1 mL of colloidal gold solution was added first with 6 µL of 0.2 mol /L K_2_CO_3_ buffer and then 40 µg/mL N2 antigen, mixed thoroughly, and blocked with 1% BSA. After high-speed centrifugation, the precipitate was suspended in 100 µL of phosphate buffer solution (PBS). Afterwards, colloidal gold resuspension solution was gold-sprayed with 0.5 µL/mm gold, dried at 37 °C for 2 h, and fixed onto the conjugate pad. Then, 1 mL of colloidal gold solution was first added with 4 µL of 0.2 mol /L K_2_CO_3_ buffer and then 20 µg/mL chicken IgY, blocked with 1% BSA, and centrifuged at high speed to obtain the precipitate. The resultant precipitate was suspended in 100 µL of PBS. Afterwards, the resuspension solution was gold-sprayed with 0.5 µL/mm gold, dried at 37 °C for 2 h, and fixed onto the conjugate pad.

N2 protein and goat anti-chicken IgY solution were streaked and coated on the nitrocellulose (NC) membrane at 1 mg/mL and 0.5 mg/mL, respectively, and dried to obtain the NC membrane containing a T line and a C line. The absorbent filter paper, the nitrocellulose (NC) membrane containing the T line and C line, a colloidal-gold-labeled N2 protein conjugate pad, a treated sample pad, and a polyvinyl chloride (PVC) bottom plate were assembled in sequence, pressed, and added with a desiccant to obtain the colloidal gold immunochromatographic strip.

**Figure 3:**
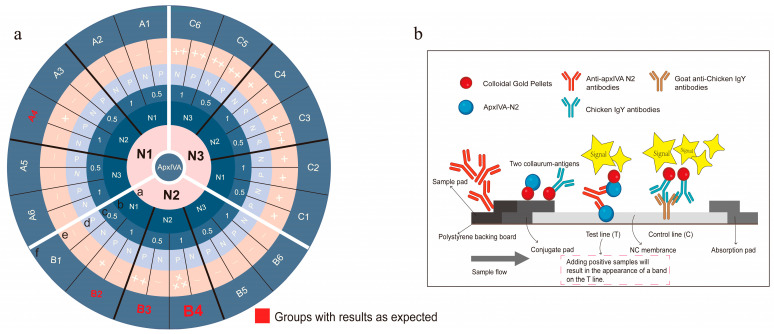
Preparation of colloidal gold immunochromatographic strip. (**a**) Screening of the optimal colloidal-gold-coupled proteins. Ring a, the colloidal-gold-coupled proteins on the conjugate pad; Ring b, the proteins on the T line; Ring c, concentration of the proteins on the T line; Ring d, P represents positive serum, N represents negative serum; Ring e, “+” indicates a positive result, and “−” indicates a negative result; Ring f, naming of the combinations. (**b**) Schematic of the strip structure.

Screening of colloidal-gold-coupled proteins. The colloidal-gold-labeled protein on the conjugate pad and the protein coated on the T line on the NC membrane were screened according to orthogonal experiments. The concentration of the protein coated on the T line was set as 0.5 mg/mL and 1 mg/mL. The specific grouping is shown in Figure 3a.

Preparation of monoclonal antibodies. The monoclonal antibodies (mAbs) against these proteins were prepared according to our previously reported method [21]. Briefly, 6- to 9-week-old female SPF BALB/c mice purchased from the Laboratory Animal Center of Huazhong Agricultural University (Wuhan, China) were immunized with 50 μg purified protein at 2-week intervals after the protein and Freund’s adjuvant were emulsified at a 1:1 volume ratio. Three immunizations were followed by a final booster injection. In the first immunization, the mice were injected with proteins emulsified with complete Freund’s adjuvant; in the second and third immunizations, they were injected with proteins emulsified with an incomplete Freund’s adjuvant; and in the final immunization, they were injected with the proteins alone. Then, mouse splenocytes were harvested and fused with SP2/0 cells using PEG4000. The positive hybridoma cells screened using ELISA and Western blot were cloned by a limiting dilution. After three rounds of limiting dilution, the 10^5^–10^6^ hybridoma cells secreting antibodies stably were injected into liquid-paraffin-pretreated abdomen cavities of BALB/c mice. On day 10, after hybridoma cell injection, the ascites containing mAbs were harvested and analyzed by ELISA, Western blot, and other methods. The selected mAbs were validated using the method we previously reported, including titration assays, chromosomal analysis, and specificity testing (Western blot and ELISA) [21]. All the animal experiments were approved by the Animal Experimental Ethical Inspection of Laboratory Animal Centre, Huazhong Agriculture University (Wuhan, China; identification code: HZAUMO-2025-0005).

Evaluation of colloidal gold immunochromatographic strip. A total of 30 GDV serum samples were used to evaluate the performance of the test strips. The diagnostic sensitivity, diagnostic specificity, PPV, NPV, and 95% CI of the colloidal gold immunochromatographic strip were calculated in the same way as for the N2 ELISA. For details, please refer to the “Evaluation of N2 ELISA” section in the Materials and Methods. The number of biological replicates for this assay is detailed in the Results section.

Analytical methods. The data processing and analyses were performed using Microsoft Office 2016.

## 3. Results

Preliminary truncation and expression of apxIVA. After bioinformatics analysis of the hydrophilicity, hydrophobicity (Figure 2b), transmembrane structural domains (Figure 2c), antigenic epitope prediction, and three-dimensional structure prediction (Figure 2d) of apxIVA, the apxIVA was first truncated into three segments, N1, N2, and N3, with a size of 918 bp, 756 bp, and 1035 bp, respectively (Figure 2a). The size of the PCR amplification products of N1, N2, and N3 segments was consistent with expectations, indicating successful amplification (Figure 2e). The pET-30a vector, which carries only small molecular tags (His tag, 0.86 kDa, and S tag, 2 kDa), was selected for recombinant protein expression. The sodium dodecyl sulfate–polyacrylamide gel electrophoresis (SDS-PAGE) results demonstrated that N1, N2, and N3 were successfully expressed and purified (Figure 2f), with the N2 protein being solubly expressed (42 kDa); the N1 and N3 proteins were inclusion bodies at 43 and 45 kDa, respectively. The Western blot results confirmed correct expression of the N1, N2, and N3 proteins (Figure 2g).

Secondary truncation and expression of apxIVA. To maximize immunogenicity, the N2 segment was extended, while maintaining its soluble expression. Three segments were selected and named N2a2, N2b2, and N2c2 (Figure 2a). The size of each amplified segment was as expected, suggesting PCR amplification was successful (Figure 2h). Only N2c2 was solubly expressed, whereas N2a2 and N2b2 were inclusion bodies (Figure 2f,g).

Evaluation of immunogenicity and distinguishing performance of candidate proteins. The results indicated that N2c2 was close to N2 in immunogenicity, but N2 was better able to distinguish APP-infected pig sera from the sera of pigs vaccinated with the APP GDV than N2c2 (Table 1). Therefore, the N2 protein was selected as the best candidate, and the optimal conditions for the N2 ELISA were determined as follows: the protein encapsulation concentration was 0.625 µg/mL; the sera to be tested were diluted at 1:20 and incubated at 37 °C for 30 min; goat anti-pig IgG (HRP) was diluted at 1:5000 and incubated at 37 °C for 30 min; and 3,3′,5,5′-tetramethylbenzidine (TMB) was incubated at 20–25 °C (room temperature) in the dark for 10 min.

Similarly, the cut-off value, specificity, sensitivity, reproducibility, and shelf-life of the N2-based ELISA method were evaluated. The cut-off value was calculated as 0.3500, based on 15 sera from pigs vaccinated with the APP GDV (Table S11). The N2-based ELISA could detect positive sera up to a 160-fold dilution, exhibiting good specificity (Table S12). The N2-based ELISA kit displayed an overall concordance rate of 90% with the ApxIV-ELISA Antibody Detection Kit (Wuhan Keqian Biological Co., Ltd.), with diagnostic sensitivity, diagnostic specificity, PPV, and NPV being 95.56%, 80.00%, 93.48%, and 85.71%, respectively (Tables S13 and S14). The shelf-life of the N2-based ELISA kit was ≥6 months at 4 °C. A total of 574 clinical sample sera from pig farms were detected with the N2-based ELISA kit (Table S15).

**Figure 4:**
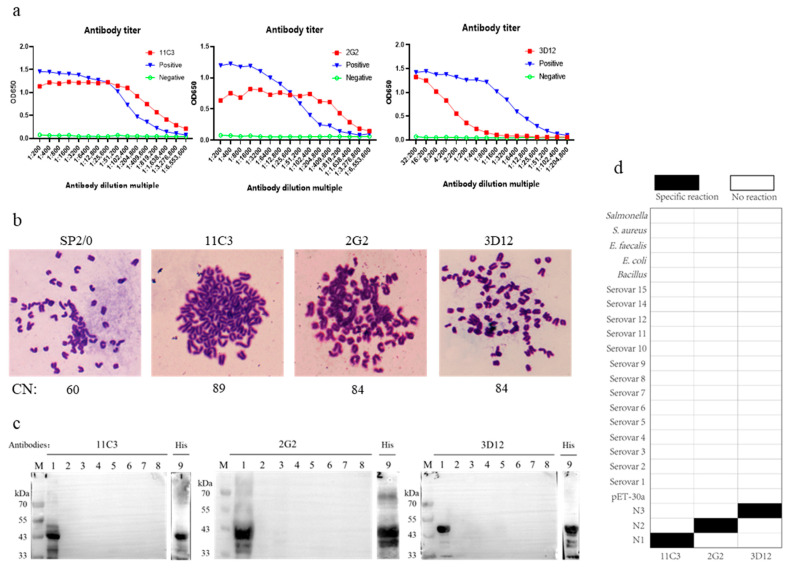
Preparation of monoclonal antibodies against apxIV protein. (**a**) Titer detection of monoclonal antibodies 11C3, 2G2, and 3D12. (**b**) Chromosome number (CN) analysis of hybridoma cells. (**c**) Western blot verification of monoclonal antibody specificity. Lane M: marker; Lane 1, 9: recombinant proteins; Lane 2: pET-30a vector bacterial lysate; Lane 3: APP 4074 strain bacterial lysate (in vitro culture); Lane 4–8: sequentially, *Bacillus* lysate, *E. coli* lysate, *E. faecalis* lysate, *S. aureus* lysate, and *Salmonella* lysate. (**d**) ELISA validation of monoclonal antibody specificity. Serovar 1-15: APP serovar 1-15 strain lysates.

Screening of colloidal-gold-coupled proteins. The orthogonal experiments were conducted to screen the optimal pairing of colloidal-gold-coupled protein with the protein on the nitrocellulose (NC) membrane (test line, T line) using pairwise combinations of the N1, N2, and N3 proteins (Figure 3a). The results indicated that when both the colloidal-gold-coupled protein and the protein on the NC membrane were an N2 protein, the T line band of the colloidal gold immunochromatographic strip was bright and clear, and the detection results were accurate. However, when the colloidal-gold-coupled protein and the protein on the NC membrane were N1 and/or N3 proteins, most of the strip detection results were false-negative or false-positive (Ring f in Figure 3a). These results may be attributed to the better fluidity of the soluble N2 protein, which could be the primary reason for the successful construction of the N2 colloidal gold immunochromatographic strip (Figure 3b). Additionally, three monoclonal antibodies, 11C3, 2G2, and 3D12, against the recombinant proteins apxIV N1, N2, and N3, respectively, were prepared (Figure 4), and these monoclonal antibodies were also of great significance in the basic research and diagnostic tool development for APP. However, when goat anti-chicken lgY polyclonal antibody was placed on the C line (control) of the NC membrane, the chromogenic bands were superior (brighter and clearer) to those when monoclonal antibody 2G2 was on the C line, which might be due to the higher overall potency of the polyclonal antibody. Furthermore, these monoclonal antibodies were used to develop APP antigen diagnostic methods, but the developed methods failed to detect natural apxIV, likely due to the low content of apxIV in the lungs and blood samples of pigs with obvious lung lesions infected by APP.

**Figure 5:**
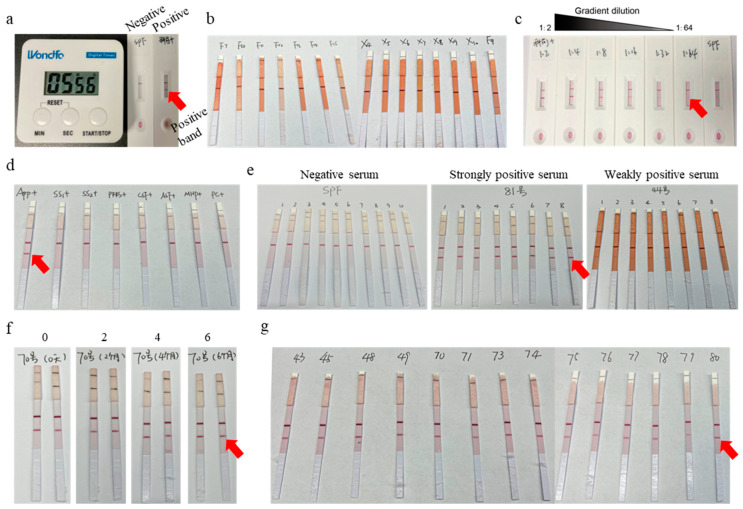
Evaluation of colloidal gold immunochromatographic strip. (**a**) Detection results of positive and negative sera. (**b**) Detection results of sera from pigs vaccinated with APP GDV. (**c**) Evaluation of sensitivity. (**d**) Evaluation of specificity. (**e**) Evaluation of reproducibility. (**f**) Evaluation of shelf-life. Lane 0: the strips stored at 4 °C for 0 months; Lane 2: the strips stored at 4 °C for 2 months; Lane 4: the strips stored at 4 °C for 4 months; Lane 6: the strips stored at 4 °C for 6 months. Each test was performed in duplicate. (**g**) Clinical sample test results (partial results shown).

Evaluation of colloidal gold immunochromatographic strip. The sera to be tested were diluted at a ratio of 1:2 and dropped onto the test strip. After a 5–10 min reaction, the strips with positive sera showed clear bands (Figure 5a), while those used to test the negative sera (specific pathogen free, SPF) and APP GDV sera displayed no bands (Figure 5b). The maximum dilution fold of positive sera that could be detected by the strip was 1:64, which was defined as the sensitivity of the strip (Figure 5c). The sera infected with APP, *S. suis*, porcine reproductive and respiratory syndrome virus (PRRSV), swine fever virus (CSFV), African swine fever virus (ASFV), *M. hyopneumoniae*, and porcine circovirus type 3 (PCV3) were used for the evaluation of strip specificity. The results showed that only positive sera of pigs infected with APP were detected, indicating the desirable specificity of the strip (Figure 5d). SPF pig sera, weakly positive sera, and strongly positive sera of APP were tested with the same batch of strips 8–10 times to evaluate the stability of the strips. The results revealed that the strips had desirable reproducibility (Figure 5e). Additionally, to evaluate the strip shelf-life, the strips stored for six months were used for positive serum detection. The data showed that the detection functions of strips stored at 4 °C remained stable for at least 6 months after; thus, the shelf-life of the strips was determined to be 6 months or more (Figure 5f). A total of 100 clinical serum samples were used to examine the detection concordance rate between the N2-based strip and the ApxIV-ELISA Antibody Detection Kit. The results showed an overall concordance rate of 89% (Table 2), with the diagnostic sensitivity, diagnostic specificity, PPV, and NPV being 92.11%, 79.17%, 93.33%, and 76.00%, respectively (Table 3).

Detection of clinical samples. In total, 289 clinical serum samples from two pig farms were tested with colloidal gold immunochromatographic strips, and the results demonstrated that the positive detection rate of the farm using the APP GDV was 4.57%, with 8 positive samples and 167 negative samples in this farm. The positive detection rate of the other farm, which did not use the APP GDV, was 42.98%, with 49 positive samples and 65 negative samples (Figure 5g and Table 4).

## 4. Discussion

The prevention and control of APP pose significant challenges for both small- and large-scale pig farms. Understanding the apxIV proteins shared by different serotypes of APP contributes to the basic research and diagnosis of APP. In this study, a 756 bp truncated protein, apxIVA N2, was screened after a six-segment truncation. This protein is structurally and functionally closer to the natural apxIV protein as it contains only a single His (0.86 kDa) tag and a single S (2 kDa) tag. We developed two antibody detection methods, namely, indirect ELISA and colloidal gold immunochromatographic strips, based on the N2 protein, and our data show that both of them demonstrated an excellent detection performance. These two methods were used for successfully detecting wild-strain-infected samples and distinguishing them from the samples of pigs vaccinated with the subunit vaccine or the APP GDV.

Natural apxIV protein is secreted in low amounts only after pigs are infected with APP, making the isolation of natural apxIV protein and related research difficult. It is difficult to completely express recombinant apxIV protein (5418 bp) due to its long sequence. When the *apxIV* gene was first discovered, apxIV plasmids were constructed to express recombinant proteins [18]. As expected, these recombinant apxIV proteins were inclusion bodies. After the initial validation of these inclusion bodies, such as the validation of their cross-reactivity with different serotypes of *A. pleuropneumoniae* antibodies, ELISA assays were developed using these inclusion bodies, and some of them are still being used in China today. According to a previous report that the N-terminus of apxIVA has higher immunogenicity than its C-terminus [22], we screened the truncated apxIVA proteins from the N-terminus. Our apxIVA N-terminal truncation experiments indicated that a 756 bp apxIVA N2 (2401–3156 bp) sequence was expressed solubly in the absence of a large tag. The N2 protein was selected as the best candidate, primarily due to its superior distinguishing performance. Since soluble proteins tend to have more specific binding sites and a lower background, N2 could be a suitable research target. Moreover, N2 carries fewer and smaller tags, and thus the spatial structure and function of this protein are closer to those of the natural protein, implying that research data based on the N2 protein are more reliable, especially in studies of molecule interaction mechanisms. Some researchers have attempted to remove tags using protein cleavage techniques, but the success rate is low, and the cleavage efficiency is also poor (based on our experience with His tag cleavage). Additionally, there is a potential risk of structural damage to the target protein after cleavage. Therefore, selecting small-molecular-weight tags before expression can effectively avoid these issues. In addition, we stopped the translation of the C-terminal His-tag of the pet-30a vector with the termination codon TAA. It should be noted that the maximal OD_630_ value of the apxIVA N2 ELISA kit is lower than that of some outer-membrane-protein-based ELISA kits, which might be due to the low secretion amount of apxIV and its potent antibody, or the short operation time in this study. Our screened 2G2 monoclonal antibody exhibited a low OD_630_ value. Notably, the OD_630_ value of 2G2 remained unchanged when it was serially diluted to 1: 409,600 (Figure 4a). In spite of the less satisfactory maximal OD_630_ value of our N2-based ELISA method, animal diagnosis was performed after the occurrence of symptoms, when the apxIV antibody secreted in pigs was sufficient to reach the minimum detection limit of our method. We plan to further optimize the reaction conditions and improve the reagents in future studies to increase the OD value and improve sensitivity and overall detection performance. Using our method, it took only 30 min for both primary and secondary antibodies to be incubated, which is more valuable than improving the maximal Od_630_ value. Extending the apxIVA truncation sequence is an effective way to improve immunogenicity, and thus we extended the truncated apxIVA sequence and obtained N2c2. Although both N2 (756 bp) and N2c2 (1083 bp) were solubly expressed, N2c2 exhibited a slightly higher immunogenicity than N2, but unlike N2, N2c2 failed to distinguish samples infected by the wild-strain APP from those vaccinated with the APP GDV, and therefore apxIVA N2 was selected for subsequent colloidal gold development. In the follow-up studies, the immunogenicity of the N2 protein will be further improved, and meanwhile, its function of distinguishing different samples should be retained.

Although the colloidal gold immunochromatographic strip method is relatively mature, no commercial strips for APP detection are available on the market, which might be due to the lack of suitable proteins. Our developed N2 strip is the first one to be used in the clinic. Our orthogonal experiments on N1, N2, and N3 proteins demonstrated that the strips constructed with N2 protein exhibited higher detection accuracy, while those constructed with N1 and N3 proteins showed more false-positive or false-negative results, which might be attributed to the advantages of N2 soluble protein over N1 and N3 inclusion bodies, but the specific mechanism remains to be explored. In the concordance tests and clinical experiments, the selected serum samples met the requirements for blinding and randomization, and we were unaware of the true background of each serum sample. In the calculation of diagnostic metrics, we temporarily treated the results from the commercial ELISA kit to determine the true positivity and negativity of these serum samples. Based on this, we calculated the diagnostic sensitivity, diagnostic specificity, NPV, and PPV for our ELISA method and the colloidal gold immunochromatographic strip method. It should be noted that this approach may cause the detection performance of our method to appear lower than it actually is.

Traditional apxIVA-based ELISA may fail to distinguish APP-infected samples from the samples of pigs vaccinated with the APP GDV (APP-HB-04M, 2022, New Veterinary Drug Certificate No. 73), as the apxIV gene is partially retained in the APP GDV. In 2004, the ELISA kit was first constructed based on the inclusion body of apxIVA N-terminal [23], and it took 135 min to perform diagnostic manipulation using this ELISA kit. Our APP detection method developed based on the soluble protein N2 can distinguish samples infected with APP from those vaccinated with the subunit vaccine or the APP GDV, with a diagnostic manipulation time of only 75 min. In order to obtain accurate results, the details of the ELISA experiments need to be emphasized, especially the reagent temperature and time control when adding samples. It should be noted that increasing the reagent temperature and extending the time spent in sample addition meant increasing the antibody incubation temperature and time, thus raising the OD_630_ value in the ELISA experiment. A change in reagent temperature in the case of direct sunlight, air conditioning, and other causes should not be ignored, especially during large-scale, long-time sample addition. The above issues should be given importance, especially in TMB incubation. Therefore, the procedures and reaction temperatures in the manual should be strictly followed so as to ensure the accuracy of the experiment results.

Some new APP detection strategies have been proposed, but their application is challenging due to their high cost and instability. For example, Sarkar et al. (2022) developed a new isothermal polymerase chain reaction method, in which the reactants are mixed with a dye, emitting a yellow-green fluorescence for APP detection, but their method has not been adopted by pig farms due to the high reagent price and false-positive rate [24]. In addition, some multichannel detection methods have been developed. For example, a multiplexed liquid array platform developed by Giménez-Lirola LG et al. (2014) can simultaneously detect four antibodies: apxI, apxII, apxIII, and apxIV [25]. Although our previous study found that the multiplex PCR method was less satisfactory due to the presence of the non-target bands caused by primer-to-primer mismatches, it is worthwhile to develop multiplex methods for the detection of respiratory pathogens in pigs. Furthermore, qPCR methods have gained popularity due to their high sensitivity and specificity in detecting APP in various samples, such as nasal swabs and lung homogenates [26,27]. However, the high cost and complexity of qPCR equipment and reagents still pose significant barriers to widespread adoption in field applications [28]. Considering the limitations in experimental conditions and available resources, we did not choose to compare the APP molecular diagnostic method in this study, primarily because we only collected serum samples, which are not suitable for the molecular detection of APP. Proper sample sources for molecular detection include nasal swabs, lung homogenates, and isolated strains.

In conclusion, a 756 bp apxIVA N2 truncated protein was screened after six-segment truncation, and this N2 protein carries only one His (0.86 kDa) tag and one S (2 kDa) tag, making it close to the natural apxIV protein in structure and function. Based on this N2 protein, we developed two antibody detection methods, indirect ELISA and colloidal gold immunochromatographic strips, and these two methods could successfully detect wild-strain-infected samples and distinguish them from the samples of pigs vaccinated with the subunit vaccine or the APP GDV (APP-HB-04M). The screening of the apxIVA N2 truncated protein and the development of two N2-based APP detection methods are of great significance for basic research on APP, the differential diagnosis of vaccine strains, and pig control breeding, exhibiting great application potential in the on-site diagnosis of APP, particularly in remote areas lacking detection instruments and professionals.

## Figures and Tables

**Table 1:** Evaluation of the distinguishing performance of N1, N2, N3, and N2c2 ELISA.

Sample No.	Serum Types *	ELISA Methods			
		N1	N2	N3	N2c2
1	APP-positive	0.750	0.773	0.829	0.759
2	SPF-antibody-negative	0.050	0.050	0.050	0.050
3	APP-HB-04M-positive (collected 21 d after the second immunization of 70-day-old pigs)	0.308	0.118	0.356	0.234
4		0.525	0.191	0.535	0.256
5		0.692	0.230	0.680	0.386
6		0.576	0.150	0.482	0.232
7		0.420	0.139	0.365	0.275
8		0.539	0.156	0.459	0.184
9		0.512	0.282	0.550	0.264
10		0.562	0.263	0.493	0.310
11		0.407	0.133	0.412	0.139
12		0.452	0.265	0.493	0.301
Distinguishing performance		no	yes	no	yes

* APP, Actinobacillus pleuropneumoniae (APP).

**Table 2:** Comparison with commercial ELISA kit.

Products *	Sample Number	Positive Number	Negative Number	Consistent Samples Number	Concordance Rate
N2 strip	100	75	25	11	89%
Commercial kit	100	76	24		

* N2 strip, the APP colloidal gold immunochromatographic strip; commercial kit, the ApxIV-ELISA Antibody Detection Kit (Wuhan Keqian Biological Co., Ltd.).

**Table 3:** Diagnostic accuracy evaluation metrics of N2 strip.

Diagnostic Metrics *	Value (%)	95%CI (%)
Diagnostic Sensitivity	92.11	85.74–98.48
Diagnostic Specificity	79.17	62.37–95.97
PPV	93.33	87.31–99.35
NPV	76.00	58.40–93.60

* Due to the unclear background of the serum samples from pig farms, the diagnostic metrics of N2 strips were calculated using the commercial kit results as the reference standard.

**Table 4:** Summary of sample detection results with N2 strip.

Sample Source	Sample Number	Vaccination or Not *	Positive Number	Negative Number	Detection Rate
Pig Farm in Guangxi Province, China	175	Yes	8	167	4.57%
Pig Farm in Hubei Province, China	114	No	49	65	42.98%
Total	289		57	232	24.57%

* Yes, samples vaccinated with *apxIV*-partially deleted vaccine. No, samples vaccinated without gene-deleted vaccine.

## Data Availability

The original contributions presented in this study are included in the article/Supplementary Materials. Further inquiries can be directed to the corresponding author(s).

## Supplementary Materials

The following supporting information can be downloaded at: https://www.mdpi.com/article/10.3390/vetsci12030278/s1, Table S1: Primers used for PCR; Table S2: Determination of the optimal encapsulation concentration (EC) and serum dilution folds of N1 ELISA; Table S3: Determination of the optimal encapsulation concentration (EC) and serum dilution folds of N2 ELISA; Table S4: Determination of the optimal encapsulation concentration (EC) and serum dilution folds of N3 ELISA; Table S5: Determination of the optimal encapsulation concentration (EC) and serum dilution folds of N2c2 ELISA; Table S6: Determination of optimal blocking solution types for N1, N2, N3, N2c2 ELISA; Table S7: Determination of optimal dilution solution types for N1, N2, N3, N2c2 ELISA; Table S8: Determination of optimal sample incubation time for N1, N2, N3, N2c2 ELISA; Table S9: Determination of optimal incubation time of HRP-conjugated secondary antibody for N1, N2, N3, N2c2 ELISA; Table S10: Determination of optimal TMB incubation time for N1, N2, N3, N2c2 ELISA; Table S11: Results for 15 sera from pig vaccinated with gene-deleted vaccine; Table S12: N2 ELISA sensitivity test; Table S13: Comparison with commercial ELISA kit; Table S14: Diagnostic Accuracy Evaluation Metrics of N2 ELISA; Table S15: Summary of sample detection results.
