# Supplementary material for: Novel Soluble apxIVA-Truncated Protein and Its Application to Rapid Detection and Distinction of Actinobacillus pleuropneumoniae Wild-Strain-Infected Samples from Those Vaccinated with apxIV-Partially Deleted Vaccine
Source: Vet Sci. 2025 Mar 16;12(3):278. doi: 10.3390/vetsci12030278 (PMC11946594; doi:10.3390/vetsci12030278)
Supplement: Supplementary file 1 [file vetsci-12-00278-s001.zip › vetsci-3413258-supplementary.pdf]

Supplemental Materials

Novel Soluble apxIVA-Truncated Protein and Its Application to Rapid Detection and Distinction of *Actinobacillus pleuropneumoniae* Wild-Strain-Infected Samples from Those Vaccinated with *apxIV*-Partially Deleted Vaccine

Table S1. Primers used for PCR.

Strain (GenBank)	Amplified fragments	Direction	Target gene	Sequence (5′ to 3′)	Product size，bp	Temp	Location (position)
APP serovar 1 str. 4074 (AF021919)	N1	Forward<br>Reverse	*ApxIV*	CCGGAATTCATGCGCGCCTATATCTGGAAT<br>CCGCTCGAGTTTTATTTCTTCTTTCGTTATGTACTCGCT	918	55°C	1543-2460
	N2	Forward<br>Reverse	*ApxIV*	CCGGAATTCATGGAGAACCTGTACTTCCAAGGGCCTAAGGCGGATCCTAAGCGG<br>CCGCTCGAGTTACCAGCCCGTTGCGGTACGAAT	756	55°C	2401-3156
	N3	Forward<br>Reverse	*ApxIV*	CCGGAATTCATGGGGCGACAAGGCGCGTTATTC<br>CCGCTCGAGTTTCCCTTCGAATTGTTTCGCATTAACGC	1035	50°C	3097-4131
	N2a2	Forward<br>Reverse	*ApxIV*	CCGGAATTCATGGACCCATCCGGTATCGGTGGAACGGTAAA<br>CCGCTCGAGTTACGTTGCCGCCCATTTATCTAAAATGGCAG	2046	57°C	1726-3771
	N2b2	Forward<br>Reverse	*ApxIV*	CCGGAATTCATGGGCACTAAAATCACCCGTAGGATTGCGG<br>CCGCTCGAGTTAGCCCATTTGTGCAAAAGTACCGTCCG	1545	57°C	1993-3537
	N2c2	Forward<br>Reverse	*ApxIV*	CCGGAATTCATGCCCTTAGCCCCTTACACTAAAAATGGCGTGG<br>CCGCTCGAGTTAGGCTAATGTCGCAAAACCGTGTGCAG	1083	57°C	2209-3291

Supplemental Table S2 Determination of the optimal encapsulation concentration (EC) and serum dilution folds of N1 ELISA

**EC μg/mL**	**P/N**	**Serum dilution folds**					
		**1: 20**	**1: 40**	**1: 80**	**1: 160**	**1: 320**	**1: 640**
	+	0.923	0.719	0.473	0.384	0.227	0.167
0.625	-	0.055	0.059	0.058	0.058	0.053	0.054
	P/N	16.782	12.186	8.155	6.621	4.283	3.093
	+	1.036	0.717	0.525	0.386	0.248	0.180
1.25	-	0.058	0.057	0.058	0.058	0.057	0.058
	P/N	17.862	12.579	9.052	6.655	4.351	3.103
	+	0.977	0.721	0.589	0.388	0.241	0.179
2.5	-	0.064	0.063	0.064	0.069	0.055	0.056
	P/N	15.266	11.444	9.203	5.623	4.382	3.196
	+	0.963	0.749	0.517	0.391	0.236	0.179
5	-	0.055	0.059	0.057	0.057	0.058	0.068
	P/N	17.509	12.695	9.070	6.860	4.069	2.632
	+	1.008	0.732	0.527	0.388	0.243	0.175
10	-	0.058	0.059	0.059	0.060	0.069	0.057
	P/N	17.379	12.407	8.932	6.467	3.522	3.070
	+	0.868	0.760	0.540	0.341	0.220	0.167
20	-	0.057	0.056	0.054	0.061	0.067	0.058
	P/N	15.228	13.571	10.000	5.590	3.284	2.879
	+	0.906	0.773	0.535	0.375	0.242	0.171
40	-	0.060	0.064	0.067	0.067	0.070	0.062
	P/N	15.100	12.078	7.985	5.597	3.457	2.758
	+	0.806	0.622	0.473	0.359	0.219	0.168
80	-	0.060	0.064	0.062	0.067	0.067	0.067
	P/N	13.433	9.719	7.629	5.358	3.269	2.507

Supplemental Table S3 Determination of the optimal encapsulation concentration (EC) and serum dilution folds of N2 ELISA

EC μg/mL	P/N	Serum dilution folds					
		1: 20	1: 40	1: 80	1: 20	1: 320	1: 640
0.625	+	1.013	0.981	0.910	0.759	0.623	0.481
	-	0.038	0.046	0.047	0.047	0.047	0.048
	P/N	26.658	21.326	19.362	16.149	13.255	10.021
1.25	+	1.019	0.976	0.890	0.729	0.607	0.457
	-	0.048	0.047	0.048	0.050	0.047	0.049
	P/N	21.229	20.766	18.542	14.580	12.915	9.327
2.5	+	1.087	0.963	0.916	0.725	0.586	0.419
	-	0.048	0.047	0.048	0.046	0.048	0.050
	P/N	22.646	20.489	19.083	15.761	12.208	8.380
5	+	1.044	0.950	0.878	0.733	0.582	0.443
	-	0.050	0.049	0.048	0.048	0.049	0.047
	P/N	20.880	19.388	18.292	15.271	11.878	9.426
10	+	1.081	0.941	0.837	0.734	0.594	0.429
	-	0.050	0.048	0.049	0.047	0.047	0.049
	P/N	21.620	19.604	17.082	15.617	12.638	8.755
20	+	1.015	0.929	0.839	0.703	0.540	0.384
	-	0.049	0.048	0.049	0.049	0.048	0.052
	P/N	20.714	19.354	17.122	14.347	11.250	7.385
40	+	1.039	0.864	0.840	0.703	0.541	0.422
	-	0.050	0.054	0.055	0.049	0.051	0.053
	P/N	20.780	16.000	15.273	14.347	10.608	7.962
80	+	1.057	0.927	0.803	0.725	0.563	0.408
	-	0.050	0.048	0.049	0.052	0.050	0.052
	P/N	21.140	19.313	16.388	13.942	11.260	7.846

Supplemental Table S4 Determination of the optimal encapsulation concentration (EC) and serum dilution folds of N3 ELISA

EC μg/mL	P/N	Serum dilution folds					
		**1: 20**	**1: 40**	**1: 80**	**1: 160**	**1: 320**	**1: 640**
0.625	+	1.072	1.063	1.107	1.082	1.096	1.058
	-	0.075	0.066	0.067	0.064	0.061	0.062
	P/N	14.293	16.106	16.522	16.906	17.967	17.065
1.25	+	1.005	1.034	0.973	1.01	1.009	1.05
	-	0.065	0.057	0.052	0.052	0.062	0.057
	P/N	15.462	18.140	18.712	19.423	16.274	18.421
2.5	+	1.005	0.994	0.948	0.956	0.966	1.015
	-	0.069	0.063	0.062	0.063	0.056	0.053
	P/N	14.565	15.778	15.290	15.175	17.250	19.151
5	+	0.99	0.989	0.97	0.935	0.954	0.954
	-	0.74	0.066	0.067	0.054	0.062	0.056
	P/N	1.338	14.985	14.478	17.315	15.387	17.036
10	+	0.998	0.976	0.971	0.937	0.965	0.947
	-	0.07	0.063	0.06	0.064	0.057	0.054
	P/N	14.257	15.492	16.183	14.641	16.930	17.537
20	+	1.085	1.083	1.027	0.962	1.032	0.953
	-	0.072	0.077	0.07	0.057	0.068	0.058
	P/N	15.069	14.065	14.671	16.877	15.176	16.431
40	+	1.088	1.044	1.083	1.103	1.082	1.046
	-	0.087	0.08	0.083	0.073	0.07	0.073
	P/N	12.506	13.050	13.048	15.110	15.457	14.329
80	+	1.156	1.162	1.177	1.164	1.191	1.131
	-	0.115	0.106	0.092	0.083	0.092	0.081
	P/N	10.052	10.962	12.793	14.024	12.946	13.963

Supplemental Table S5 Determination of the optimal encapsulation concentration (EC) and serum dilution folds of N2c2 ELISA

EC μg/mL	P/N	Serum dilution folds					
		**1: 20**	**1: 40**	**1: 80**	**1: 160**	**1: 320**	**1: 640**
0.625	+	0.939	0.952	0.952	0.929	0.908	0.889
	-	0.046	0.053	0.054	0.046	0.053	0.049
	P/N	20.413	17.962	17.630	20.196	17.132	18.143
1.25	+	1.024	1.007	0.963	0.996	0.914	0.924
	-	0.052	0.054	0.044	0.048	0.043	0.043
	P/N	19.692	18.648	21.886	20.750	21.256	21.488
2.5	+	1.137	1.076	1.028	0.957	0.97	0.937
	-	0.058	0.054	0.056	0.044	0.053	0.048
	P/N	19.603	19.926	18.357	21.750	18.302	19.521

5	+	1.05	1.072	1.045	0.972	1	0.934
	-	0.054	0.065	0.057	0.046	0.047	0.062
	P/N	19.444	16.492	18.333	21.130	21.277	15.065
10	+	0.993	1.019	0.983	0.96	0.886	0.851
	-	0.059	0.063	0.061	0.056	0.054	0.065
	P/N	16.831	16.175	16.115	17.143	16.407	13.092
20	+	1.04	0.928	0.937	0.976	0.854	0.829
	-	0.135	0.125	0.043	0.056	0.05	0.071
	P/N	7.704	7.424	21.791	17.429	17.080	11.676
40	+	1.089	1.031	0.989	0.964	0.954	0.874
	-	0.067	0.056	0.064	0.055	0.048	0.054
	P/N	16.254	18.411	15.453	17.527	19.875	16.185
80	+	1.092	1.113	1.025	1.007	0.952	0.854
	-	0.063	0.065	0.065	0.064	0.053	0.059
	P/N	17.333	17.123	15.769	15.734	17.962	14.475

Supplemental Table S6 Determination of optimal blocking solution types for N1, N2, N3, N2c2 ELISA

P/N	Blocking solution types															
	1%BSA				2%BSA				1%BSA				5% skim milk			
	N1	N2	N3	N2c2	N1	N2	N3	N2c2	N1	N2	N3	N2c2	N1	N2	N3	N2c2
+	0.981	0.821	0.975	0.901	0.965	0.891	0.995	0.915	0.945	0.882	0.987	0.894	0.903	0.804	0.927	0.890
-	0.055	0.048	0.061	0.057	0.052	0.047	0.061	0.057	0.052	0.048	0.061	0.057	0.051	0.048	0.060	0.056
P/N	17.84	17.10	15.98	15.81	18.56	18.96	16.31	16.05	18.17	18.38	16.18	15.68	17.71	16.75	15.45	15.89

Supplemental Table S7 Determination of optimal dilution solution types for N1, N2, N3, N2c2 ELISA

P/N	Dilution solution types															
	PBST				2%BSA				PBST				5% skim milk			
	N1	N2	N3	N2c2	N1	N2	N3	N2c2	N1	N2	N3	N2c2	N1	N2	N3	N2c2
+	1.035	0.971	1.05	1.020	0.950	0.887	0.991	0.913	0.955	0.893	0.972	0.904	0.920	0.850	0.913	0.870
-	0.057	0.053	0.066	0.064	0.050	0.047	0.060	0.056	0.052	0.048	0.061	0.057	0.051	0.048	0.060	0.056
P/N	18.16	18.32	15.91	15.94	19.00	18.87	16.52	16.30	18.37	18.60	15.93	15.86	18.04	17.71	15.22	15.54

Supplemental Table S8 Determination of optimal sample incubation time for N1, N2, N3, N2c2 ELISA

P/N	Sample incubation time											
	15min				30min				60min			
	N1	N2	N3	N2c2	N1	N2	N3	N2c2	N1	N2	N3	N2c2
+	0.392	0.36	0.45	0.328	0.963	0.889	0.994	0.916	1.053	0.935	1.104	0.955
-	0.048	0.05	0.052	0.048	0.049	0.048	0.058	0.056	0.058	0.052	0.061	0.057
P/N	8.17	7.20	8.65	6.83	19.65	18.52	17.14	16.36	18.16	17.98	18.10	16.75

Supplemental Table S9 Determination of optimal incubation time of HRP-conjugated secondary antibody for N1, N2, N3, N2c2 ELISA

P/N	Incubation time of HRP-conjugated secondary antibody											
	15min				30min				60min			
	N1	N2	N3	N2c2	N1	N2	N3	N2c2	N1	N2	N3	N2c2
+	0.456	0.428	0.494	0.423	0.944	0.832	0.994	0.915	1.053	0.897	1.097	0.955
-	0.048	0.047	0.05	0.048	0.052	0.048	0.058	0.056	0.061	0.061	0.061	0.061
P/N	9.50	9.11	9.88	8.81	18.15	17.33	17.14	16.34	17.26	14.70	17.98	15.66

Supplemental Table S10 Determination of optimal TMB incubation time for N1, N2, N3, N2c2 ELISA

P/N	TMB incubation time											
	5min				10min				15min			
	N1	N2	N3	N2c2	N1	N2	N3	N2c2	N1	N2	N3	N2c2
+	0.61	0.532	0.67	0.595	0.953	0.825	1.032	0.91	0.996	0.831	1.035	1.04
-	0.048	0.048	0.048	0.048	0.052	0.051	0.052	0.054	0.058	0.058	0.058	0.058
P/N	12.71	11.08	13.96	12.40	18.33	16.18	19.85	16.85	17.17	14.33	17.84	17.93

Supplemental Table S11 Results for 15 sera from pig vaccinated with gene-deleted vaccine

Sample No.	OD_{630}	Sample No.	OD_{630}	Sample No.	OD_{630}
1	0.291	6	0.265	11	0.175
2	0.313	7	0.166	12	0.230
3	0.173	8	0.331	13	0.189
4	0.282	9	0.191	14	0.284
5	0.263	10	0.199	15	0.171

Supplemental Table S12 N2 ELISA sensitivity test

Serum dilution folds	OD_{630}
1:20	0.814
1:40	0.586
1:80	0.471
1:160	0.351
1:320	0.211
1:640	0.131

Supplemental Table S13 Comparison with commercial ELISA kit

Kits*	Sample number	Positive number	Negative number	Consistent samples number	Concordance rate
N2 ELISA kit	60	46	14	54	90%
Commercial kit	60	45	15		

*Commercial kit, ApxIV-ELISA Antibody Detection Kit (Wuhan Keqian Biological Co., Ltd.).

Supplemental Table S14 Diagnostic Accuracy Evaluation Metrics of N2 ELISA

Diagnostic Metrics*	Value (%)	95%CI (%)
Diagnostic Sensitivity	95.56	88.46-100.00
Diagnostic Specificity	80.00	61.20-98.80
PPV	93.48	85.55-100.00
NPV	85.71	66.31-100.00

*Due to the unclear background of the serum samples from pig farms, the diagnostic metrics of N2 ELISA were calculated using the commercial kit results as the reference standard.

Supplemental Table S15 Summary of sample detection results

Sample source	Sample number	Vaccination or not*	Positive number	Negative number	Detection rate
Pig Farm in Guangxi Province, China	385	Yes	14	371	03.64%
Pig Farm in Hubei Province, China	189	No	55	134	29.10%
Total	574		69	505	12.02%

*Yes, samples vaccinated with *apxIV*-partially deleted vaccine; No, samples vaccinated without gene deleted vaccine.
